# Electrode perforation of a silicone breast implant in a subcutaneous implantable cardioverter-defibrillator carrier: A previously unreported device complication

**DOI:** 10.1016/j.hrcr.2026.07.018

**Published:** 2026-07-30

**Authors:** Theodoros Maximidou, Jonas Wörmann, Wolfram Malter, Daniel Steven, Jakob Lüker

**Affiliations:** 1 Department of Electrophysiology, Heart Center, Medical Faculty and University Hospital Cologne, University of Cologne, Cologne, Germany; 2 Department of Gynecology and Gynecologic Oncology, Medical Faculty and University Hospital Cologne, University of Cologne, Cologne, Germany

**Keywords:** Subcutaneous implantable cardioverter-defibrillator, Device complication, Parasternal electrode, Breast implant, Implant rupture, Siliconoma, Ulcerative mastitis

**Key Teaching Points**

• A subcutaneous implantable cardioverter-defibrillator (S-ICD) electrode can perforate submuscular silicone breast implants, leading to extracapsular silicone migration, foreign-body granuloma (siliconoma) formation, and ulcerative mastitis.
• A regular S-ICD interrogation does not exclude a mechanical complication. Impedance may remain stable if the defibrillation electrode stays predominantly subcutaneous, and the absence of overt over- or undersensing does not rule out electrode dislodgement. Where available, comparison of stored sensing-vector electrograms with prior interrogations may help reveal subtle changes related to altered chest-wall anatomy or electrode position.
• Pre-procedural screening for breast implants is essential in all female patients considered for S-ICD implantation. Implant type, anatomical plane (subpectoral vs subfascial), and a focused physical examination of the operative site should be documented. Cross-disciplinary discussion can address alternative device or tunneling strategies when anatomical conflict is anticipated.
• In newly diagnosed potentially reversible nonischemic cardiomyopathy, premature permanent ICD implantation should be avoided. Current European Society of Cardiology guidelines recommend at least 3 months of optimized guideline-directed medical therapy with repeat left ventricular ejection fraction assessment before primary-prevention ICD implantation.
• Patient education post-implantation should address warning symptoms such as persistent chest-wall pain, breast asymmetry, swelling, or skin changes. When these symptoms arise, contrast-enhanced computed tomography may provide valuable diagnostic information, while avoiding susceptibility artifacts that might limit cardiac magnetic resonance imaging interpretation in S-ICD carriers.

## Introduction

Subcutaneous implantable cardioverter-defibrillators (S-ICDs) eliminate intravascular lead-related risks but require parasternal and lateral electrode tunneling.^1^ Submuscular breast implants alter chest wall anatomy, particularly through expansion of the lower breast pole.^2^ Whether these subfascial anatomy alterations carry the risk of conflict between the S-ICD electrode trajectory and the implant capsule has not been investigated yet.

We report a case of a parasternal S-ICD electrode perforation into a submuscular silicone breast implant, resulting in extracapsular silicone migration, foreign-body granuloma (siliconoma) formation, and ulcerative mastitis in a 42-year-old woman (Clinical timeline shown in Figure 1 and Table 1). A comprehensive search of PubMed, Embase, and Web of Science (inception to June 26, 2026) identified no previous reports describing this complication. Systematic search terms were defined by 2 researchers, using Boolean operators combining specific terms related to this case with terms for defibrillators, breast prostheses, and electrode-related events. Titles and abstracts were screened for any report of interaction between an S-ICD and a breast implant. The full search strategy, including the search string, can be found in the Supplemental Table 1. Prior publications have reported S-ICD generator migration with impedance changes and shock delivery failure,^3^ transvenous ICD generator migration into a ruptured breast implant pocket,^4^ or breast asymmetry,^5^ but direct interaction of the S-ICD electrode with a silicone breast implant has not been reported to date.

**Figure 1:**
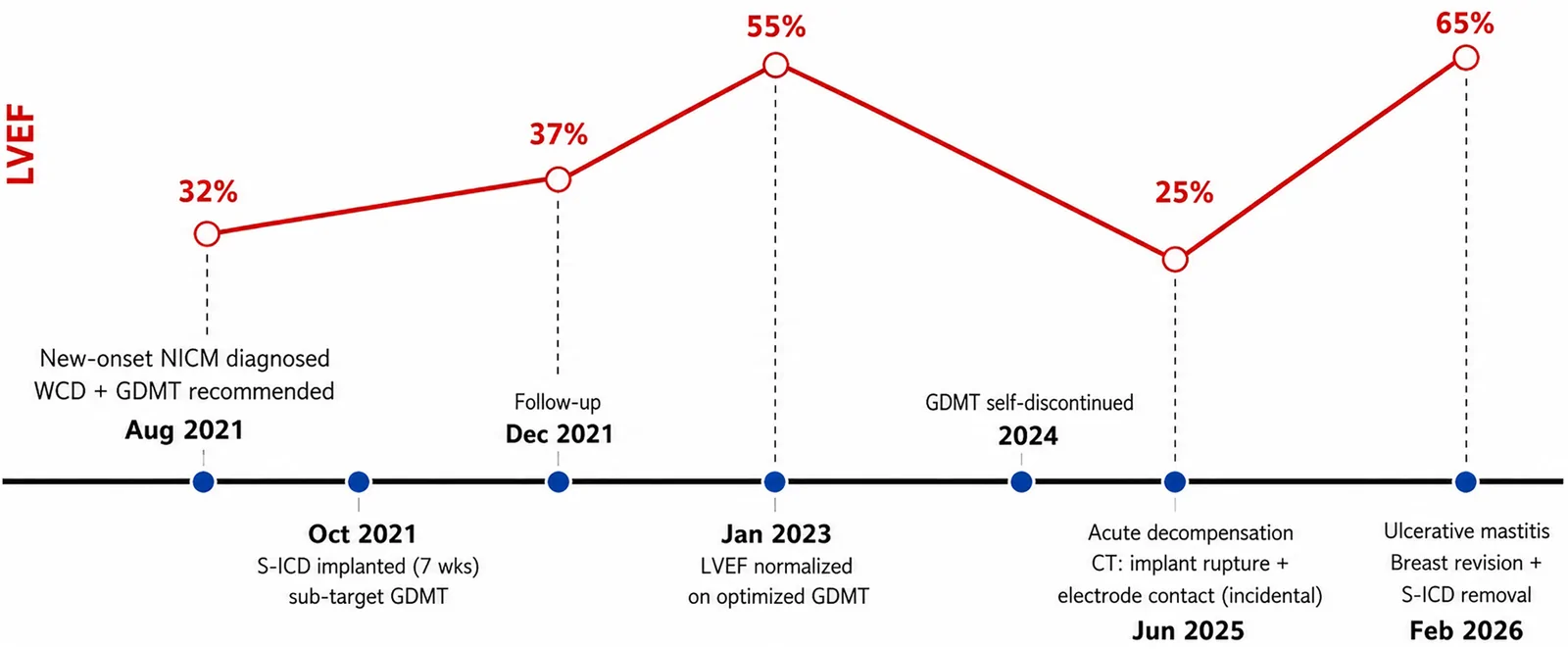
Clinical timeline. CT = computed tomography; GDMT = guideline-directed medical therapy; LVEF = left ventricular ejection fraction; NICM = nonischemic cardiomyopathy; S-ICD = subcutaneous implantable cardioverter-defibrillator; WCD = wearable cardioverter-defibrillator.

**Table 1:** Clinical timeline and echocardiographic follow-up

**Clinical event**	Aug 2021	Dec 2021	Jan 2023	Jun 2025	Feb 2026
	NICM diagnosis (S-ICD implant on October 13, 2021)	Follow-up	Follow-up	Acute decompensation∗	Breast revision and S-ICD removal
**LVEF (%)**	32	37	55	25	65
**LVEDD (mm)**	66	60	49	67	42
**NT-proBNP (ng/L)**	2611	—	163	4295	362
**GDMT status**	Initiated	Sub-target	Optimized	Discontinued†	Reinstated

GDMT = guideline-directed medical therapy; LVEDD = left ventricular end-diastolic diameter; LVEF = left ventricular ejection fraction; NICM = nonischemic cardiomyopathy; NT-proBNP = N-terminal pro-B-type natriuretic peptide; S-ICD = subcutaneous implantable cardioverter-defibrillator.

∗ Acute decompensation occurred after delivery of third child (March 2025).

† Patient self-discontinued all heart failure medications in early 2024.

This report describes a previously unrecognized complication of S-ICD electrode tunneling and proposes practical recommendations for preventive measures. As a secondary observation, the case illustrates the clinical relevance of device timing in newly diagnosed nonischemic cardiomyopathy (NICM).

## Case presentation

A 42-year-old woman presented to the gynecology department of our institution in February 2026 with an ulcerative lesion on the left breast (Figure 2A). Clinical examination and ultrasound confirmed ulcerative mastitis. Her medical history included bilateral submuscular silicone breast augmentation, and she had been receiving guideline-directed medical therapy (GDMT) for idiopathic NICM with a prior left ventricular ejection fraction (LVEF) of 32%. An S-ICD (Boston Scientific, EMBLEM™ S-ICD) had been implanted for primary prevention at another center in 2021, but this information was not noted in the patient history at this point. A current preoperative transthoracic echocardiography showed heart failure with improved ejection fraction (HFimpEF; LVEF 65%).

**Figure 2:**
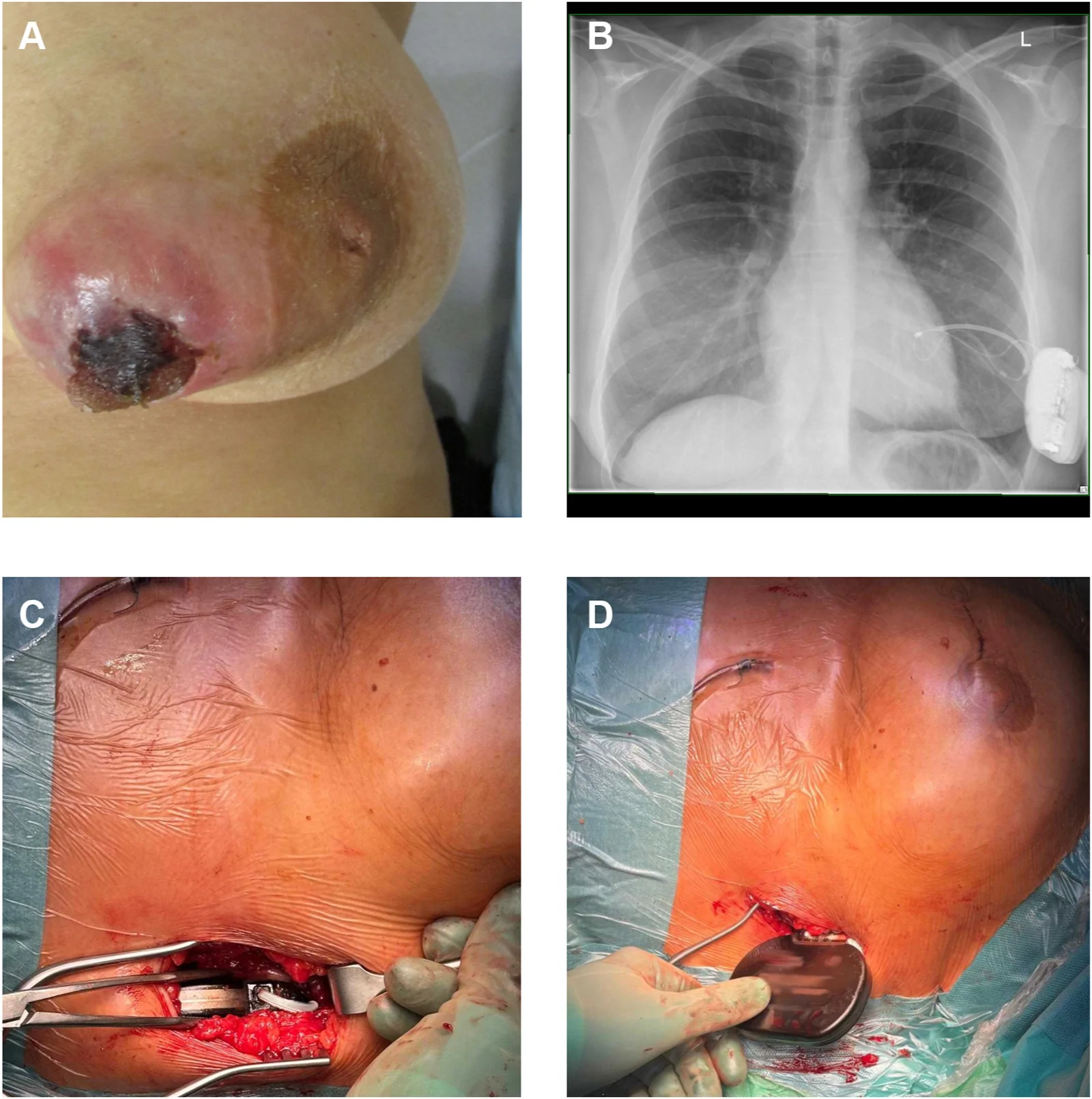
Clinical photographs. **A:** Ulcerative lesion on left breast. **B:** Radiograph after breast revision surgery. **C and D:** Explantation of subcutaneous implantable cardioverter-defibrillator generator and electrode via anterior axillary incision.

Surgical revision of the ulcerative lesion confirmed rupture of the left silicone implant. Excision of the abscess cavity revealed foreign material embedded within inflamed granulomatous tissue. This was identified as the parasternal subcutaneous defibrillation electrode of the S-ICD system. After breast implant removal, the device and lead were left *in situ*, and the electrophysiology department was consulted. Histopathology confirmed extensive foreign-body granulomas with giant-cell reaction, without evidence of malignancy. Microbiological cultures were negative.

Retrospective case investigation revealed that the patient had first presented to another hospital in August 2021 with exertional dyspnea and reduced exercise tolerance (New York Heart Association functional class II–III). Laboratory workup showed markedly elevated N-terminal pro–B-type natriuretic peptide (NT-proBNP) (2611 ng/L). Echocardiography at that time demonstrated severely reduced LVEF with global hypokinesia and a dilated left ventricle (LVEDD, 66 mm). Coronary angiography excluded obstructive coronary artery disease, and endomyocardial biopsy showed no evidence of active myocarditis or detectable viral genome. The diagnosis of new-onset NICM of uncertain etiology was established. The discharge summary documented recommendations for wearable cardioverter-defibrillator bridging and stepwise GDMT optimization, with echocardiographic reassessment of LVEF before consideration of permanent ICD implantation. However, an S-ICD was implanted at the same institution 7 weeks later (October 13, 2021). GDMT was still at sub-target doses (sacubitril/valsartan 24/26 mg, bisoprolol 2.5 mg, and spironolactone 25 mg). The presence of bilateral submuscular breast implants was not noted in the implantation operative report. A reassessment of LVEF before implantation was not documented. Follow-up echocardiography in December 2021 showed an improvement in LVEF (37%), and by January 2023, LVEF had normalized to 55% under optimized GDMT, with an improved LVEDD of 49 mm. NT-proBNP had decreased to 163 ng/L.

In early 2024, the patient discontinued GDMT without seeking medical consultation. In June 2025, she was hospitalized with signs of congestive heart failure. Clinical examination revealed volume overload and a body weight of 103 kg. Transthoracic echocardiography showed severely reduced LV function (LVEF 25%) with functional mitral regurgitation (vena contracta 7 mm), severe tricuspid regurgitation, and biatrial dilatation. NT-proBNP was markedly elevated at 4295 ng/L. High-dose intravenous diuretic therapy was initiated. However, laboratory testing showed rising creatinine and lactate levels consistent with low-output syndrome, requiring administration of levosimendan. After hemodynamic stabilization, the patient was successfully decongested, GDMT was re-established, and she was discharged with a weight of 90 kg. A contrast-enhanced chest computed tomography (CT), performed at admission to exclude pulmonary embolism, incidentally demonstrated rupture of the left breast implant with direct S-ICD electrode contact (Figure 3A–3D). However, in the context of the acute cardiac decompensation, this finding was not pursued further. A cardiac magnetic resonance imaging (MRI) did not show late gadolinium enhancement but was of limited diagnostic value due to extensive susceptibility artifacts from the S-ICD. With continued adherence to optimized GDMT, LVEF recovered again and reached 65% by February 2026.

**Figure 3:**
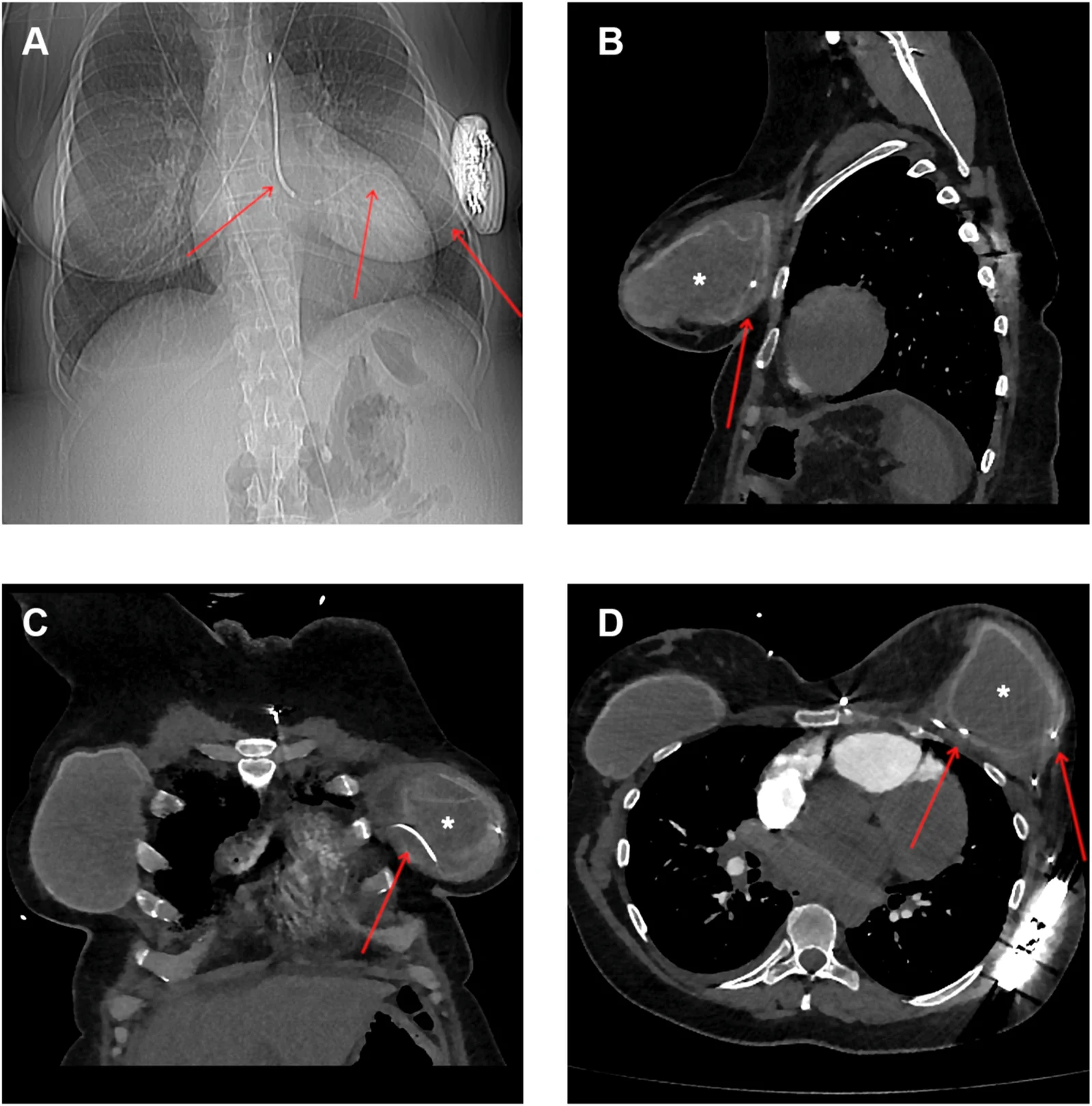
Computed tomography of the thorax. *Asterisks (∗)* indicate breast implants; *red arrows* indicate the electrode. **A:** Frontal topogram. **B:** Sagittal reconstruction. **C:** Frontal reconstruction. **D:** Transversal reconstruction.

Between discharge in mid-2025 and readmission in early 2026, the patient developed ulcerative mastitis prompting the breast surgery described earlier. Interrogation of the S-ICD following breast surgery showed no malfunction with an impedance of 56 Ω and with no oversensing or undersensing in the programmed secondary sensing vector configuration. Over 4.3 years, zero therapies had been delivered, and no episodes of ventricular tachyarrhythmia had been detected. During breast surgery, the fixation suture had detached, and the electrode had dislocated into the implant cavity, confirmed by radiography (Figure 2B). It was decided to only use an anterior axillary line incision for surgical access. Electrode extraction required only gentle manual traction, and after severing the fixation sutures, the generator was removed (Figure 2C and 2D). There was no significant bleeding. Given the sustained LVEF recovery, the absence of arrhythmic events, and shared decision-making, no reimplantation was indicated, and the patient was discharged 1 day after S-ICD system extraction. Outpatient follow-up visits were uneventful.

## Discussion

This case illustrates a previously unreported complication of S-ICD implantation in patients with breast implants, as the S-ICD electrode and submuscular silicone breast implants showed direct interaction. Review of the chest CT imaging (Figure 3) demonstrates the electrode trajectory passing through the ruptured silicone implant. On the contrast-enhanced study, the electrode could be followed from its parasternal course into the implant capsule. There was no evidence of an alternative cause of rupture of the breast implant. It seems likely that implant rupture was caused by direct penetration during the implantation procedure. However, we cannot exclude the possibility that chronic repetitive pressure by the electrode eroded the silicone capsule over time. The available imaging does not allow the 2 mechanisms to be distinguished with certainty. No chest imaging predating the June 2025 CT study was available to date the onset of rupture. In the original surgical report of the S-ICD implantation, the pre-existing breast implant was not acknowledged, and, likely, it was not accounted for during implantation.

S-ICD electrode placement involves subcutaneous tunneling from the xiphoid to the lateral device pocket using a rigid tunneling tool with a peel-off sheath.^6^ The electrode trajectory courses caudal to the pectoral muscle in the subfascial plane. Breast prostheses are typically placed subpectoral or subfascial.^7^ It cannot be ruled out that cranial deviation of the tunneling trajectory during implantation may result in direct penetration of breast prostheses, particularly when implanters are unaware of their presence. According to the available operative report from the implanting institution, the S-ICD was implanted using an incision-based technique guided by anatomical landmarks without fluoroscopy. The report did not document the exact tunneling plane or any assessment of the breast implant. Detailed operator experience and procedural information beyond the available operative report could not be retrieved for this retrospective analysis.

Although previous reports primarily describe generator-related complications with breast implants,^1^, ^2^, ^3^, ^4^, ^5^ this case extends the spectrum to include electrode-mediated implant rupture with significant tissue consequences. The chronic foreign-body reaction to extracapsular silicone with giant-cell reaction (siliconoma) preceded clinical detection, although this incidental finding remained unnoticed at first. The American College of Radiology recommends MRI as the imaging modality of choice to detect silicone breast implant-related complications.^8^ In this case, the contrast-enhanced CT imaging was diagnostically sufficient and free from susceptibility artifacts that limited cardiac MRI interpretation. Importantly, standard device interrogation parameters remained normal throughout. Even after breast surgery with electrode dislodgment, impedance (56 Ω; 1 year prior: 60 Ω) and sensing remained stable, highlighting that electrical parameters are not sufficient to reliably detect every mechanical complication. Notably, impedance may remain largely unaffected because the defibrillation electrode remains predominantly within the subcutaneous space despite implant perforation. Likewise, the absence of overt over- or undersensing does not exclude a mechanical complication. However, because electrode dislodgment alters the position of the sensing electrodes relative to the cardiac signal, all 3 sensing vectors (primary, secondary, and alternate) would have been expected to show a change in electrogram morphology. The primary and secondary sensing vectors might also have been sensitive to chest-wall geometry, since they incorporate the generator in their sensing vector. The alternate vector, which is derived between the distal tip and the proximal ring of the parasternal electrode, might not be affected by tissue changes between generator and electrode. Unfortunately, this could not be assessed retrospectively, as stored electrograms predating electrode migration were unavailable. Where available, comparison of stored sensing-vector electrogram morphologies with prior device interrogations may, therefore, provide additional evidence of subtle changes related to altered chest-wall anatomy or electrode position.

To prevent similar occurrences, we propose systematic pre-procedural screening. Female patients considered for S-ICD implantation should be questioned about breast augmentation history, with documentation of implant type and anatomical plane. When breast implants are identified, a focused physical examination of the intended operative site should be performed, and pre-procedural imaging (CT, MRI, or ultrasound) should be considered. If anatomical conflict is anticipated, alternative strategies should be discussed through shared decision-making, including modified electrode tunneling (within device geometry constraints), transvenous ICD systems, or an extravascular ICD (Aurora EV-ICD, Medtronic)^9^ or breast implant revision or removal before S-ICD implantation. It is important to mention that the relative merits of these alternatives in patients with breast implants have not been studied directly. Since comparative data are lacking, the choice should be individualized. Intraoperative documentation is equally critical. Awareness of breast implants should be noted in operative reports. Although slight caudal electrode positioning might theoretically reduce direct implant contact, fixed geometry and 45 cm length of S-ICD electrodes limit flexibility, and any deviation must maintain appropriate sensing vectors and defibrillation efficacy.^7^ Sex-specific considerations are relevant in this context. In a survey of female S-ICD recipients, 44% expressed a preference for a more cranial and posterior generator position due to bra-related discomfort.^10^ Regarding pre-operative preparation before breast surgery, a focused physical examination of the planned surgical site should be performed to identify relevant anatomical variants, pathologies, or implanted medical devices.

Post-implantation surveillance should include patient education regarding symptoms (persistent pain, breast changes, and device pocket swelling) and low-threshold imaging for clinical suspicion. This case also shows that cross-specialty communication is essential. Breast surgeons should acknowledge cardiac implantable electronic devices before implant-related procedures, and comprehensive device interrogation should be performed before any surgical intervention.^11^

As a secondary observation, this case also illustrates challenges of ICD indication for new-onset NICM. Current European Society of Cardiology guidelines recommend ≥3 months of optimized GDMT with LVEF reassessment before primary-prevention ICD implantation.^12^^,^^13^ In this case, S-ICD implantation occurred only 7 weeks after initial presentation with sub-target medication dosage, and without documented preoperative LVEF reassessment. Subsequent LVEF normalization demonstrates favorable treatment responsiveness on GDMT in this patient.

Recent registry data support systematic adherence to guideline-recommended waiting periods. In the SCD-PROTECT study, LVEF improved above 35% within 2 months in more than half of newly diagnosed cardiomyopathy patients managed with a wearable cardioverter-defibrillator.^14^ Notably, despite 2 episodes of severe cardiac decompensation in this patient, no ventricular arrhythmic events were documented over 4.3 years of continuous device monitoring, further underscoring the importance of guideline adherence, complete documentation, and periodic reassessment of device indication in patients with potentially reversible cardiomyopathy.

### Limitations

First, causality and the exact sequence of interaction cannot be proven from the available retrospective data. Second, as a single-case observation, the true incidence of this complication and its relationship to specific implant characteristics (subpectoral vs subfascial placement, implant volume, or implant age) remains unknown. Third, the preventive recommendations proposed here are derived from a single observation and represent expert opinion rather than evidence from comparative studies. They should be regarded as hypothesis-generating and interpreted in light of this report’s limitations.

## Conclusion

S-ICD electrode perforation into a submuscular silicone breast implant can cause significant complications. Importantly, device interrogation may show regular parameters despite significant dislocation. This case describes a previously unreported complication and suggests the need for structured screening before S-ICD implantation. Routine storage of sensing-vector electrograms may be a valuable addition to standard S-ICD follow-up, as longitudinal comparison with prior recordings could help identify subtle morphology changes associated with altered chest-wall anatomy or electrode position, even when impedance and conventional sensing parameters remain unchanged. Cross-specialty communication is required when both cardiac devices and breast implants coexist. Comprehensive perioperative device interrogation should be performed in accordance with periprocedural cardiac implantable electronic device guidance. The case also reinforces the importance of adhering to guideline-recommended LVEF reassessment before permanent ICD implantation in new-onset NICM.

## Disclosures

JW: speaker fees from Abbott and Boston Scientific, and consulting fees from AtriCure; all outside the submitted work. DS: lecture honoraria from Biosense Webster, Abbott, and Pfizer, advisory fees from Boston Scientific and Abbott, and research grants from Biosense Webster and Abbott; all outside the submitted work. JL: speaker fees from Abbott, Boston Scientific and Johnson & Johnson, and consulting fees and research funding from Boston Scientific; all outside the submitted work. All other authors report no conflicts of interest.
