# Supplementary material for: Electrode perforation of a silicone breast implant in a subcutaneous implantable cardioverter-defibrillator carrier: A previously unreported device complication
Source: HeartRhythm Case Rep. 2026 Jul 30;12(10):1240–5. doi: 10.1016/j.hrcr.2026.07.018 (PMC13687959; doi:10.1016/j.hrcr.2026.07.018)
Supplement: Supplementary file 1 [file mmc1.docx]

**Supplementary Table 1. Literature search strategy**

| **Databases** | PubMed, Embase, and Web of Science |
| --- | --- |
| **Date range** | Database inception to June 26, 2026 |
| **Language restriction** | None |
| **Search string (adapted per database syntax)** | ("ICD" OR "subcutaneous ICD" OR "S-ICD" OR "subcutaneous implantable cardioverter-defibrillator" OR "subcutaneous defibrillator") AND ("breast implant" OR "breast augmentation" OR "silicone" OR "mammoplasty" OR "breast*") AND ("perforation" OR "erosion" OR "migration" OR "rupture" OR "complication") |
| **Fields searched** | Title, abstract, and keywords |
| **Inclusion criteria** | Reports (case reports, case series, registries, or cohort studies) describing any interaction between an S-ICD system and a breast implant |
| **Exclusion criteria** | Articles without an S-ICD–breast-implant interaction |
| **Records identified** | 237 |
| **After duplicate removal** | 111 |
| **Excluded (title/abstract)** | 102 |
| **Full-text assessed** | 9 |
| **Reports of S-ICD electrode perforation of a breast implant** | 0 |
| **Related reports identified** | - ICD generator-related complications (e.g., generator migration with impedance change and shock failure) - Coexistence of an ICD generator and a breast implant (e.g. a transvenous-ICD generator near a ruptured implant pocket - Planned or combined ICD implantation with breast augmentation/implant revision - Device or lead migration (non S-ICD) toward or into breast tissue (mimicking breast cancer, or lead migration to the breast after cardiac perforation) - Esthetic and anatomic considerations in female CIED recipients |

**Abbreviations:** CIED = cardiac implantable electronic device; ICD = implantable cardioverter-defibrillator; S-ICD = subcutaneous implantable cardioverter-defibrillator.
